# Mitigation of salt stress in wheat seedlings by halotolerant bacteria isolated from saline habitats

**DOI:** 10.1186/2193-1801-2-6

**Published:** 2013-01-11

**Authors:** Dhanushkodi Ramadoss, Vithal K Lakkineni, Pranita Bose, Sajad Ali, Kannepalli Annapurna

**Affiliations:** Division of Microbiology, Indian Agricultural Research Institute, New Delhi, 110012 India

**Keywords:** Halotolerant bacteria, Salt stress, Wheat, Plant growth promoting bacteria

## Abstract

Eighty four halotolerant bacterial strains were isolated from the saline habitats and screened for growth at different NaCl concentrations. All grew well at 5% NaCl, but only 25% isolates showed growth at 20% NaCl concentration. Five strains SL3, SL32, SL35, J8W and PU62 growing well in 20% NaCl concentrations were further characterized for multiple plant growth promoting traits such as indole −3- acetic acid (IAA) production, HCN and siderophore production, ACC deaminase activity and P-solubilization. None were positive for HCN production and PCR amplification of *acd*S, the structural gene for ACC deaminase enzyme was found negative. 16S rRNA gene sequencing analysis of the five strains showed them to belong to two genera *Bacillus* and *Hallobacillus*. *In vitro* experiments showed that salt concentrations had significant inhibitory effects on development of seedlings but not on the growth of the bacterial strains. Inoculation of the 5 halotolerant bacterial strains to ameliorate salt stress (80 mM, 160 mM and 320 mM) in wheat seedlings produced an increase in root length of 71.7% in comparison with uninoculated positive controls. In particular, *Hallobacillus* sp. SL3 and *Bacillus halodenitrificans* PU62 showed more than 90% increase in root elongation and 17.4% increase in dry weight when compared to uninoculated wheat seedlings at 320 mM NaCl stress indicating a significant reduction of the deleterious effects of NaCl. These results indicate that halotolerant bacteria isolated from saline environments have potential to enhance plant growth under saline stress through direct or indirect mechanisms and would be most appropriate as bioinoculants under such conditions.

## Introduction

Salinity is one major limiting factor to plant growth and crop productivity [1]. Cultivated soils worldwide are becoming more saline from marginal irrigation water, excessive fertilization, and desertification processes. Currently, more than 800 million hectares of land throughout the world are affected by levels of salt that could substantially reduce crop productivity [2]. Strategies for alleviation of salt stress involve developing salt-resistant cultivars, leaching excess soluble salts from upper to lower soil depths, flushing soils that contain soil crusts at the surface, reducing salt by harvesting salt-accumulating aerial plant parts in areas with negligible irrigation water or rainfall for leaching, and amelioration of saline soils under cropping and leaching [3]. An alternative is to alleviate salt stress by inoculating crop seeds and seedlings with plant growth promoting bacteria (PGPB). Looking into the perspectives of crop production losses due to the severity of abiotic stresses, especially salinity, tolerance to stress provided by microbial inoculants becomes more important. Beneficial effect of PGPB under salinity has been related to hydraulic conductance, osmolyte accumulation, sequestering toxic Na^+^ ions , maintaining higher stomatal conductance and photosynthetic activities [4].

*Azopsirillum halopraeferens*, tolerates 3% NaCl [5], colonizes mangrove roots in seawater [6], and enhances the growth of halophytes irrigated with seawater [7]. Most other *Azospirillum* species can tolerate only 2% NaCl. Similarly, some *Bacilli* strains able to tolerate 8% NaCl and showing PGP activities have been reported by Upadhyay et al. [8]. PGPB with ACC deaminase activity have been successfully used to reduce the negative effects of salinity by lowering stress ethylene production in the vicinity of growing root [9–12]. Mayak et al. [9] reported that ACC deaminase producing salt tolerant bacteria can survive well in a saline environment and that their beneficial properties help plants to overcome stress effects. Halotolerant bacteria are a group of bacteria able to grow well in media containing a wide range of NaCl (1-33%) or in the absence of NaCl [13]. Hence, it was hypothesized that isolating bacteria with PGP activities from naturally saline habitats would give bonafide candidates which could help ameliorate saline stress effect on wheat plants. Microorganisms surviving at extreme environmental conditions have been found suitable for use in different agricultural practices [14].

Wheat (*Triticum aestivum* L.) is a major staple food crop for more than one third of the world population and is the main staple food of Asia [15]. Seed germination and seedling growth of wheat, like other crops, were negatively affected by drought and salinity stresses [16]. Salinity stress adversely affects total dry matter and plant growth, as most of the energy available is used in to make osmotic adjustments by the plant [17]. The screening of salt tolerant lines/cultivars has been attempted by many researchers on various species at seedling growth stage [18]. Both water availability and salt stress can impair coleoptiles growth, thus affecting seedling establishment in the field [19, 20]. The relation of various seedling growth parameters like early emergence and fast seedling growth to seed yield and yield components under saline conditions are important for the development of salt tolerant cultivar for production under saline soil condition. The use of rhizobacteria is one of the most acceptable approaches to reduce the effect of salt-stress on plants as they are endowed with mechanisms which either modulate or ameliorate the salt stress [4, 21]. Wheat is grown both as winter and spring crop in India and is cultivated under rainfed conditions where precipitation is less than 850 mm annually. Salinity is one of the major constraints which hamper wheat production in the country. Hence, the present study was conducted in an attempt to isolate and characterize halotolerant bacteria from saline habitats e.g. Sambhar salt lake, Jaiselmer saline soils and Pushkar lake and evaluate check their ability to ameliorate saline stress and improve seedling germination of wheat under salt affected conditions.

## Results

### Isolation and screening of salt tolerant bacteria

**Figure 1:**
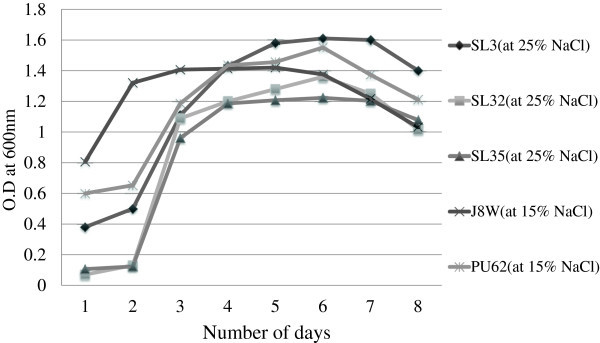
**Growth curve of selected isolates at different NaCl concentration.** The graph shows the maximum growth of each isolates in their respective tolerance levels of NaCl. Each point is the mean of three replicates.

Eighty four bacterial strains were selected based on distinct morphology on nutrient agar (NA) (1% NaCl) plates. Colonies were selected based on colour, shape, size and abundance. These were screened for salt tolerance and growth in nutrient broth amended with various concentrations of NaCl. Growth was measured after every 24 hours till the seventh day. All grew well in 5% NaCl. 24 isolates from Pushkar lake, 29 isolates from Sambhar lake and 6 isolates from Jaiselmer soil grew well in 10% NaCl, 4 isolates from Pushkar lake, 19 isolates from Sambhar lake and 4 isolates from Jaiselmer soil grew well in 15% NaCl and 3 isolates from Pushkar and 16 isolates from Sambhar lake grew in 20%NaCl. There was a gradual decrease in number of isolates growing at higher salt concentrations. From the total isolates 92.8%, 70%, 32% and 25% of bacterial isolates grew well in 5, 10, 15 and 20% NaCl respectively. Isolates from soil were tolerant to maximum of 15% NaCl where as water isolates showed higher tolerance limits (Figure 1).

### Identification and phylogenetic analysis

**Figure 2:**
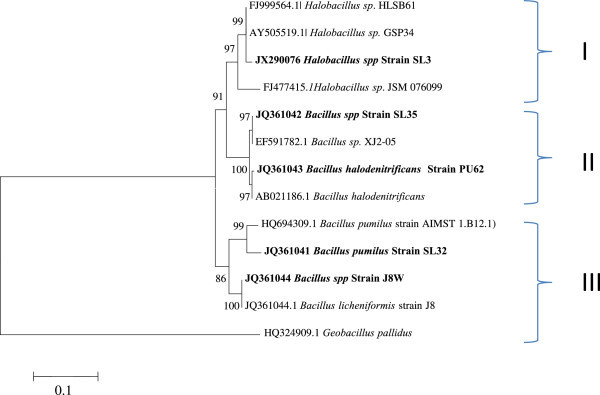
**Phylogenetic analysis of partial 16S rDNA sequences from the five selected isolates.** Bootstrap analysis was determined and values are shown. The scale bars show two substitution nucleotides per 100 nucleotides. The 16S rDNA of the reference strains were used for the tree construction and the strains mentioned in bold are isolates from this study.

The 16S rDNA of selected five halotolerant bacteria were amplified. Gel electrophoresis of undigested amplified products revealed that all isolates produced a single band of about 1500 bp. On the basis of nucleotide sequences of the 16S rDNA fragments the selected strains were identified as *Hallobacillus sp* (SL3), *Bacillus pumilus* (SL32), *Bacillus sp* (SL35), *Bacillus sp* (J8W) and*. Bacillus halodenitrificans* (PU62) (Table 1). Phylogenetic analyses of the five strains based on NJ method with 1000 boot strap sampling resulted into three cultures (Figure 2). *Hallobacillus sp*. SL3 formed cluster I, cluster II was formed with two strains *Bacillus sp* (SL35) and *Bacillus halodenitrificans* (PU62) and cluster III had *Bacillus pumilus* (SL32) and *Bacillus sp* (J8W).

**Table 1:** **Plant growth promoting traits of halotolerant bacterial isolates and their sequence identity**

Isolate*	IAA production	Siderophore production	Phosphate solubilization	HCN production	***acdS*** gene	GenBank accession number	Best matched identity >99%
SL 3	+	+	-	-	-	JX290076	*Halobacillus spp*
SL 32	-	+	-	-	-	JQ361041	*Bacillus pumilus*
SL 35	-	-	-	-	-	JQ361042	*Bacillus sp*
J 8 W	+	-	-	-	-	JQ361044	*Bacillus sp*
PU 62	-	+	+	-	-	JQ361043	*Bacillus halodenitrificans*

*Habitats: *SL*= Sambhar salt lake; *J*= Jaiselmer saline soil; *PU*= Pushkar lake.

### Characterization for PGP activities of selected strains

**Figure 3:**
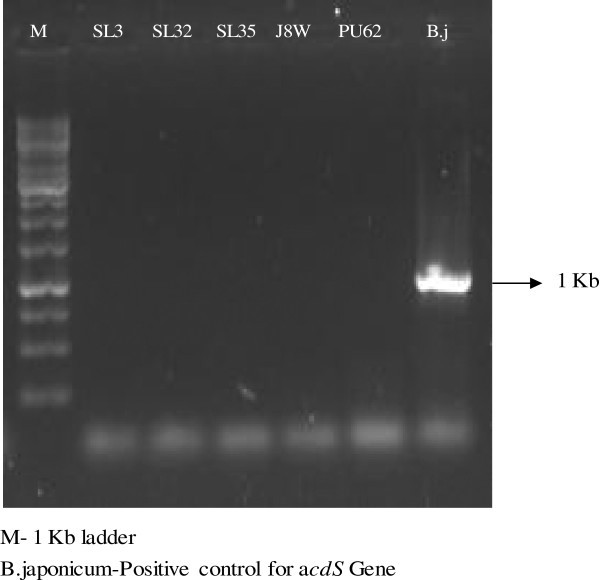
**PCR amplification of*acdS*gene shows no band from selected bacterial isolates along with the*Bradyrhizobuium japonicum*as a positive control for*acdS*gene.**

SL3 and J8W were positive for IAA production developing pink color on NA + Trp plate, PU62 solubilized insoluble form of phosphorus, e.g. tri calcium phosphate on Pikovaskya plate. Three isolates, SL3, SL 32 and PU 62 produced siderophores. An orange halo around the colony growth was observed. None of the five isolates produced HCN (Table 1). PCR amplification for *acd*S was found negative (Figure 3) and no ACC deaminase activity was observed under *in vitro* conditions in all the five isolates.

### Halotolerant bacterial inoculation effect on wheat seedlings under salt stress

**Figure 4:**
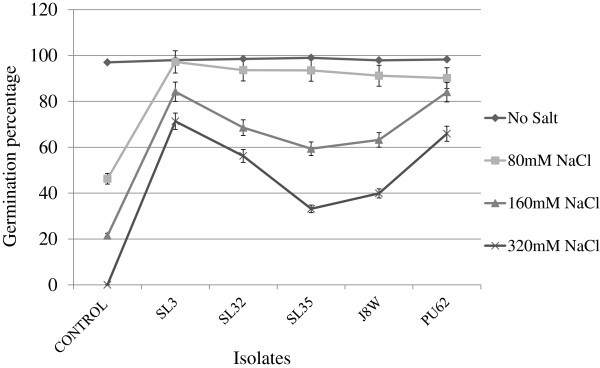
**Effects of bacterial inoculation on germination percentage of wheat seedlings at 80 mM, 160 mM and 320 mM NaCl concentration along with control (sterilized water).** Values are mean of two independent experiments of 50 seeds for each treatment.

All the five halotolerant strains were able to significantly promote the growth of wheat seedlings in the presence of salt. Salt stress (160 mM) reduced root length by 58.9% and dry weight biomass by 51% in 7 day old seedling when compared with the negative control. Inoculation *per se* had beneficial effects on the root length and root biomass. Under no salt conditions, inoculation improved root length by 11% and root biomass by 31%. All the tested halotolerant strains were able to improve root length by between 21.8% and 84.7% at 80 mM, 44% and 117% at 160 mM and 24% and 96% at 320 mM of salt stress (Table 2 ). Likewise, inoculation increased root dry weight by between 15.4% and 61.8% under salt stressed conditions in 7 day old wheat seedlings when compared with positive control (Table 3 ). Germination percent of the seed was drastically reduced under higher salt concentrations (Figure 4). There was a 54%, 79% and 100% reduction in germination at 80, 160 and 320 mM NaCl respectively under no inoculation conditions. Inoculation with halotolerant bacterial strains ameliorated the deleterious effect of NaCl by more than 50% at all the salt concentrations.

**Table 2:** **Effect of bacterial inoculation on root length (cm) of wheat under different concentrations of NaCl**

Strainsa	No Salt	80 mMNaCl	160 mMNaCl	320 mMNaCl
1	4.34±0.04^b^	4.32±0.09^a^	3.30±0.13^a^	0.96±0.04^a^
2	3.78±0.06^c^	3.81±0.05^c^	2.20±0.05^d^	0.25±0.02^d^
3	3.45±0.06^d^	2.92±0.06^d^	2.76±0.07^c^	0.57±0.03^c^
4	4.32±0.06^b^	4.09±0.05^b^	2.87±0.06^b^	0.71±0.04^b^
5	4.77±0.06^a^	4.42±0.06^a^	2.95±0.05^b^	0.89±0.03^a^
Control	3.71±0.03^c^	2.39±0.04^c^	0.52±0.03^c^	0.0^e^

Strains: 1, *Halobacillus sp* SL3; 2, *Bacillus pumilus* SL32; 3, *Bacillus sp* SL35; 4, *Bacillus sp* J8W; 5, *Bacillus halodenitrificans* PU62. *Values (mean ± SD * r=2) with the same letters are not significantly different at p ≤ 0.05. Control, sterile water. Values are mean of 50 seeds for each treatment in two independent experiments.

**Table 3:** **Effects of bacterial inoculation on root dry weight (g) of wheat under different concentrations of NaCl**

Strains	No Salt	80 mMNaCl	160 mMNaCl	320 mMNaCl
1	0.593±0.016^b^	0.585±0.01^a^	0.39±0.007^a^	0.197±0.014^a^
2	0.527±0.019^a^	0.437±0.022^b^	0.277±0.012^b^	0.117±0.016^c^
3	0.54±0.023^a^	0.475±0.031^b^	0.327±0.031^b^	0.127±0.016^c^
4	0.591±0.011^a^	0.577±0.024^a^	0.387±0.08^a^	0.18±0.009^ab^
5	0.635±0.023^a^	0.612±0.02^a^	0.387±0.08^a^	0.15±0.012^bc^
Control	0.44±0.012^b^	0.39±0.01^c^	0.22±0.01^c^	0.0^d^

Strains: 1, *Halobacillus sp* SL3; 2, *Bacillus pumilus* SL32; 3, *Bacillus sp* SL35; 4, *Bacillus sp* J8W; 5, *Bacillus halodenitrificans* PU62. *Values (mean ± SD * r=2) with the same letters are not significantly different at p ≤ 0.05. Control, sterile water. Values are mean of 50 seeds for each treatment in two independent experiments.

## Discussion

Bacteria stimulate plant growth *via* both direct and indirect mechanisms with variable results depending on a number of factors. High salinity is one of the most common environmental stress factor that adversely affect plant productivity by retarding the plant growth and development. To promote plant growth under saline condition, direct use of salt-tolerant bacteria has drawn considerable research interest both in industry and in academics. In the present study, a large number of halotolerant bacteria were isolated, and screened for their tolerance levels of NaCl. In this study, all the isolates at higher NaCl concentrations grew with long stationary phase. This could be due to the synthesis of protective factors and adaptation of current environmental conditions [22]. Phylogenetic analysis of five halotolerant bacterial 16S rDNA gene sequence revealed them to belong to *Bacillus* and *Hallobacillus* species. These strains with high salt tolerance were further characterized for the PGP activities including IAA production, P-solubilization, HCN and siderophore production and ACC deaminase activity. Upadhyay et al. [8] found that only 18% (24 out of 130) of strains isolated from wheat rhizosphere in soils of Varanasi, were found tolerant to 8% of NaCl, while maintaining PGP activities. Siddikee et al.[11] reported that different halotolerant bacteria were able to withstand high salt concentration (1.75 M NaCl) and were able to facilitate plant growth promotion in the presence of growth inhibitory levels of salt. In the present study 32% (27 out of 84) of strains could grow well at 15% NaCl and 25% (21 out of 84) at 20% NaCl. Tolerance of bacterial strains to higher salinity levels in the present study was probably because of the naturalization in the saline habitats. Five high salt tolerating bacteria were studied for their PGP activities. SL3 and J8W had multiple PGP activities (IAA and siderophore positive), however, SL32 and PU62 showed only siderophore and P-solubilization activities respectively.

PGPR that have ACC deaminase activity help plants to withstand stress (biotic and abiotic) by reducing the level of stress ethylene [9, 10]. In the present study none of the five strains studied produced ACC deaminase. Since all the five had mitigating effect of salinity stress on the wheat seedling, there must be other mechanisms by which these halotolerant bacteria are able to stimulate seed germination, root elongation and increase in root biomass. Since IAA secreted by bacteria may promote root growth directly, by stimulating cell elongation or cell division [23], the observed positive effect of strain *Hallobacillus* sp. SL3 on wheat seedling could be due to this mechanism. However, strain *B. halodenitrificans* PU62 which also enhanced these parameters could be due to some other mechanism as it did not show IAA production. It was observed that halotolerant strains, which produce IAA but not ACC deaminase, inhibited root growth rather than promote elongation in the presence of salt [11], reflecting a higher synthesis rate of ACC under stress. Alternatively, IAA concentration itself may have inhibited root growth. In our study the halotolerant bacteria producing neither IAA nor ACC deaminase enhanced root elongation and root dry weight under salt stress conditions. Also the deleterious effect of salt on seed germination rate was mitigated with inoculation. This could be the result of better water uptake induced by inoculation which is reflected in faster root growth in inoculated seedlings exposed to these stresses. A report showed that the turgor pressure at low water potential (20% PEG 6000) was higher in inoculated seedlings in two wheat cultivars under osmotic stress [24]. Another way of protection is by production of exopolysaccharides which bind with cations, including Na+, and thus decrease the content of Na+ available for plant uptake [8, 25]. Obviously, there could be more than one mechanism that PGPR employ for protection against salt induced stresses.

In this study we have shown that different halotolerant bacteria, isolated from saline habitats are able to withstand high salt concentrations and can facilitate plant growth promotion in the presence of growth inhibitory levels of salt. Given the variation in plant growth promotion, the selection and subsequent commercial use of halotolerant bacteria with multiple PGP activities can be used as bioinoculants for saline environments. This would be an important area for future research.

## Materials and methods

### Isolation of bacteria

Soil sample was collected from Jaisalmer region (26.9200° N latitude and 70.9000° E longitude) and brought to the lab under ice packs. Ten fold dilutions were made and plated on Nutrient agar (NA) medium amended with 5% NaCl. Bacteria with distinct morphology were selected, purified and maintained on slants at 4°C till further use. Bacteria isolated from water samples from Sambhar (26°54'N latitude and 75°12'E longitude) and Pushkar lakes (26.4872° N latitude and 74.5542° E longitude) of Rajasthan, India were obtained from Division of Microbiology, IARI, New Delhi.

### Screening for salt resistance

The intrinsic resistance of the bacterial isolates against salinity was evaluated by observing the growth on NA medium amended with final concentrations of NaCl (5, 10, 15, 20 and 25% (w/v)). Control plate was also maintained with 1% NaCl (w/v). The plates were incubated for 48–72 h at 28 ± 2°C . Same experiment was carried out with NaCl amended broth. Cultures grown as starters in 1% NaCl were inoculated in 5 ml tubes with different concentrations of NaCl and OD was recorded after every 24 h till 7^th^ day.

### DNA isolation and identification of salt tolerant bacteria

DNA was isolated from five high salt tolerant isolates : SL3, SL 32, SL35, J8W and PU62. Briefly, 5 ml cultures were centrifuged at 8000 rpm for 5 min. The pellet was used for DNA isolation using Zymo Fungl-Bacterial DNA kit (Zymo Research, USA) according to the manufacturer’s instruction. Amplification of 16S rDNA region was done by using universal primers Forward (5’-AGAGTTTGATCCTGGCTCAG-3’) and Reverse (5’- AAGGAGGTGATCCAGCCGCA- 3’). PCR reaction mixture was prepared by adding 30 ng of DNA, 200 μM dNTP’s, 1.5 mM MgCl_2_, 1X *Taq* buffer and 2U of *Taq* DNA polymerase. The reaction condition followed was: predenaturation at 94°C for 5 min, denaturation at 94°C for 1 min, annealing for 1 min at 57°C, extension at 72°C for 1.5 min followed by 30 cycles and final extension at 72°C for 6 min. Amplified products were analysed in 1% agarose gels. These were eluted, purified and sequenced at Sequencing Facility, Delhi University South Campus.

### Phylogenetic analysis

The 16S sequences of the five isolates were compared to 16S rDNA sequences available in the GenBank database through BLAST search. The phylogenetic analysis of the 16S rRNA gene sequence was performed using the MEGA 4.0 software package [26]. The phylogenetic tree was constructed using the neighbor joining (NJ) method [27]. The clustering stability of the tree was evaluated by bootstrap analysis of 1000 data sets. Sequence data were deposited in GenBank and accession numbers obtained.

### Characterization for plant growth promoting traits

The selected five strains were characterized for their plant growth promoting traits. Log phase cultures grown in nutrient broth supplemented with 15% NaCl were used. Siderophore production was detected by using Chrome azurol assay (CAS) developed by Schwyn and Neilands [28]. HCN production was detected by the method of Lorck [29]. The qualitative test for IAA production was carried out as given by Bric et al. [30]. Phosphorus solubilizing activity was determined according to solubilization zone assay [31]. PCR amplification of the *acd*S ; the structural gene for ACC-deaminase enzyme was done using gene specific primers [32] and the enzyme activity was determined by monitoring the amount of ammonia generated due to hydrolysis of ACC as described by Shaharoona et al. [33].

### Wheat experiment with PGPR

Log phase cultures of selected isolates SL3, SL 32, SL35, J8W and PU62 with an OD of 0.8 were used. The cultures were centrifuged at 8000 rpm for 5 min and the pellets were washed three times with sterile distilled water and suspended in 10 ml sterile water. Healthy wheat seeds var. HD 2733 were surface sterilized with 0.1%HgCl_2_ followed with washes with sterilized water for 8–10 times. Seeds were treated and imbibed in washed bacterial suspension for 30 min. For control without bacterial treatment, the surface sterilized seeds were imbibed in sterile distilled water. The imbibed seeds were placed in plates with filter paper soaked in different concentration of NaCl (80 mM, 160 mM and 320 mM). Seeds in plates treated only with water were used as negative control and seeds in plates treated only with salt solutions were used as positive control. There were 5 replications for each treatment with 10 seeds per plate. All plates were stored in dark at 15°C for 3 days and in D/N cycle (10/14 hours) for another 4 days. After 2 d, germination percent was measured and after 9 d observations for root length and dry weight were taken.

#### Statistical analysis

Data on growth parameters of wheat were subjected to analysis of variance (ANOVA). Significance at 5% level was tested by Duncan’s multiple range test (DMRT) using SAS package, Version 9.1.3 (SAS 2010).

## Purpose of the work

Very little information is available on halotolerant bacteria isolated from saline habitats with plant growth promoting activities. We wanted to evaluate these halotolerant bacteria isolated from naturally saline habitats for mitigation of deleterious effects of salt on plant growth. Such studies are necessary to select a strain to be used as bioinoculant for sustainable crop production under saline areas.
